# Reverse Pseudohyperkalemia: An Important Clinical Entity in Chronic Lymphocytic Leukemia

**DOI:** 10.1155/2015/930379

**Published:** 2015-09-27

**Authors:** Sahar Mansoor, Noa G. Holtzman, Ashkan Emadi

**Affiliations:** University of Maryland School of Medicine, Marlene & Stewart Greenebaum Cancer Center, Baltimore, MD 21201, USA

## Abstract

Hyperkalemia is a potentially lethal electrolyte derangement commonly seen in patients with hematologic neoplasms with or without renal failure. Pseudohyperkalemia and reverse pseudohyperkalemia also can be seen in this patient population and early recognition and diagnosis of these conditions are vital. Here, we report a case of reverse pseudohyperkalemia in a patient with chronic lymphocytic leukemia (CLL) and provide recommendations regarding diagnostic and therapeutic strategies for management of such patients. Further, we discuss the pathogenesis of this condition and its potential role as a surrogate of favorable prognostic features in patients with CLL.

## 1. Introduction

Hyperkalemia is a dangerous, potentially lethal electrolyte derangement. Due to its cardiotoxic properties, severe hyperkalemia can lead to cardiac myocyte hyperexcitability, dysrhythmia, and death. Increased potassium also affects the neurologic system, and patients can develop symptoms such as fatigue, muscle cramps, tetany, motor weakness, paralysis, and paresthesias. Hyperkalemia is seen frequently in renal failure and acidosis. In the cancer patient population, it serves as one of the hallmark electrolyte derangements of the feared oncologic emergency of tumor lysis syndrome. Tumor lysis syndrome results from massive lysis of cancer cells and is seen most commonly in patients with hematologic malignancies receiving chemotherapy. With lysis of cells, potassium, uric acid, and phosphorous are released into the circulation, and, among these electrolyte abnormalities, the hyperkalemia is the most immediately life threatening one. Prophylactic medications such as allopurinol and rasburicase are given to prevent the life threatening effects of tumor lysis. Every clinician caring for cancer patients is trained to closely monitor for hyperkalemia and, in such cases, to act fast by initiating potassium lowering therapies, such as calcium gluconate, insulin, albuterol, furosemide, and sodium polystyrene sulfonate and, even in emergent refractory cases, to initiate dialysis. It is thus important to recognize cases of pseudohyperkalemia or reverse pseudohyperkalemia, the latter of which is being increasingly reported in the leukemia and lymphoma population. Clinicians must be aware of these clinical entities in order to accurately diagnose and treat tumor lysis syndrome and to avoid taking measures that may cause iatrogenic hypokalemia and unnecessary harm to the patient.

## 2. Case Report

A 49-year-old man with no known past medical history presented to emergency room with 2-month history of upper respiratory symptoms, chills, dyspnea on exertion, and fatigue. Vital signs were unremarkable. Physical exam showed a well appearing male in no acute distress speaking full sentences comfortably, pale sclera, and hepatosplenomegaly without rebound or guarding. Complete blood count was notable for white blood cell count of 545 K/mcl (4.5–11), hemoglobin of 4.6 g/dL (12–17), hematocrit of 25.5% (37–50), and platelet of 47 K/mcl (153–367). Manual differential revealed 85% lymphocytes, 5% neutrophil, and no blasts. Flow cytometric analysis identified a monotypic B cell population that expressed CD5, CD19, CD20, CD22, CD23, and kappa, consistent with chronic lymphocytic leukemia (CLL). Heavy chain immunoglobulin (IgVH) mutational analysis was conducted which revealed mutated IgVH gene. A CLL fluorescence in situ hybridization (FISH) panel did not detect any chromosomal abnormalities. Comprehensive metabolic panel collected in a standard collecting vial (Light green top BD Vacutainer PST Gel and Lithium Heparin) was notable for potassium level of 9.5 mmol/L (3.5–5.1), BUN of 24 mg/dL (9–20), creatinine of 1.48 mg/dL (0.66–1.25), uric acid of 6.4 mg/dL (3.5–7.2), and lactate dehydrogenase of 1746 units/L (313–618). EKG was completely normal.

The degree of hyperkalemia was startling and inconsistent with patient's clinical status. Prior to initiation of potassium lowering therapies, potassium levels were repeated in the same collecting vial by three separate standard analyzers and resulted in similar abnormally high values. Due to the discrepancy between patient's symptoms and normal EKG with extremely high potassium levels, reverse pseudohyperkalemia in the setting of extreme leukocytosis was suspected. In order to measure whole blood (not plasma) potassium levels, a blood gas analyzing vial was used, and the vial was immediately taken to laboratory without placement of vial on ice or use of pneumonic transport. Time from specimen collection to analysis was approximately 10 minutes. Whole blood potassium returned 3.7 mmol/L. Therefore, diagnosis of reverse pseudohyperkalemia was made.

The patient was initiated on chemotherapy and whole blood potassium levels were monitored by an operating room laboratory that had the ability to analyze the whole blood potassium in a blood gas vial. Serial EKGs were obtained and all remained normal. While plasma potassium levels obtained via pneumatic transport ranged in 7.8–10.4 mmol/L during the course of therapy, manually walked plasma and whole blood potassium levels ranged in 4.2–5.5 mmol/L and 3.7–5.2 mmol/L, respectively (Table 1). Patient tolerated chemotherapy well and was discharged home without complications. This case exemplifies how recognition and diagnosis of reverse pseudohyperkalemia allowed for accurate monitoring of this patient's electrolytes for tumor lysis throughout his treatment course, which was completed.

## 3. Discussion

There are two defined clinical entities that address spurious hyperkalemia: pseudohyperkalemia and reverse pseudohyperkalemia. Pseudohyperkalemia is falsely elevated potassium in serum with normal potassium level in plasma, and the opposite is true for reverse pseudohyperkalemia, with falsely elevated potassium in plasma and normal potassium levels in serum. Plasma is usually collected in a heparinized tube, which inhibits the clotting process, while serum is collected in a tube without any other reactive agents. Common causes of pseudohyperkalemia include mechanical stressors, such as traumatic venipuncture, excess force of suctioning during blood draw, prolonged tourniquet use, contaminants, prolonged handling time, vigorous handling, and/or wrong maintenance temperature of blood specimens.

The pathogenesis of reverse pseudohyperkalemia in hematologic malignancies has not yet been delineated; however, several hypotheses exist in explaining this phenomenon. One such theory explains that the dysplastic white blood cells of leukemia and lymphoma exhibit increased fragility that makes these cells prone to lysis. Mechanical stress such as aggressive manipulation of blood tubes after drawing and delivery via a pneumatic tube transportation system that involves many abrupt accelerations, decelerations, turns, and stops can contribute to lysis of the cells and thus lead to spurious elevated potassium [1]. Another theory suggests that severe leukocytosis has higher consumption of metabolic fuels that may lead to impaired Na^+^/K^+^ ATPase pump activity, which leads to potassium release from the high number of white cells that are present in such cases [2].

The thought that reverse pseudohyperkalemia in CLL is a manifestation of increased cell membrane fragility has interesting prognostic implications. As early as 1896, Ferdinand Adolph Gumprecht observed and defined the “shadows of Gumprecht,” more commonly known today as “smudge cells” or “basket cells,” as a diagnostic microscopic feature of the malignant lymphocytes seen in CLL [3]. Smudge cells represent the irregular shape that these lymphocytes form when smeared onto a glass slide for observation under a microscope, a shape attained due to their increased membrane fragility. Several studies have shown that the percentage of smudge cells present at diagnosis of CLL serves as a prognostic indicator, with higher percentages of smudge cells associated with longer progression-free survival and overall survival [4–6]. It has also been shown that higher vimentin expression, an important cytoskeletal protein that contributes to membrane rigidity, is associated with lower percentage of smudge cells and worse prognosis in CLL [6]. Vimentin also plays a role in signal transduction and cellular activation that is thought to play a role with metastasis in solid tumors [7]. Further, CLL patients with mutated immunoglobulin heavy chain gene (IgVH), a gene whose mutation is associated with better clinical outcomes [8, 9], were found to have higher numbers of smudge cells than those with the unmutated gene [6]. An inverse relationship was also shown between smudge cell percentage and presence of CD38^+^ and ZAP-70^+^ [5], both of which are unfavorable risk factors [9]. Clearly, an interesting relationship between increased lymphocyte membrane fragility, prognosis, and clinical outcomes in CLL has been well established. As smudge cell presence is secondary to increased membrane fragility of these cells and reverse pseudohyperkalemia is another manifestation of this increased cellular susceptibility to lysis, it can be further interesting to explore if CLL patients exhibiting reverse pseudohyperkalemia at time of diagnosis have correspondingly longer overall survival. As in our patient's case of CLL, who had the IgVH mutation, it is further interesting if reverse pseudohyperkalemia is also seen in higher incidence in patients with mutated IgVH, as is the case for smudge cells, both associated with longer overall survival.

All in all, diagnosis of reverse pseudohyperkalemia in patients with hematologic neoplasms is crucial and poses a potentially interesting prognostic significance. Full time availability of laboratories that can measure whole blood potassium, at least in tertiary care cancer centers, and avoidance of utilization of pneumatic tube transport can prevent mismanagement. Early recognition can lead to the correct gentle handling of specimens and would prevent unnecessary harmful measures to be taken, as exemplified in our patient's case.

## 4. Conclusion

In all patients with significant leukocytosis from hematologic neoplasms that present with hyperkalemia, if there is no other clinical or EKG evidence of hyperkalemia, before intervening in ways that can lead to increased morbidity and mortality, early recognition and diagnosis of reverse pseudohyperkalemia are vital.

## Figures and Tables

**Table 1:** Reverse pseudohyperkalemia due to mechanical stress of specimen delivery. Potassium levels at different time points are shown, with respective LDH levels, which differ secondary to delivery mode of blood specimen for analysis.

Method of sample delivery	Day 1		Day 2		Day 3	
	Potassium (mmol/L)	LDH (units/L)	Potassium (mmol/L)	LDH (units/L)	Potassium (mmol/L)	LDH (units/L)
Pneumatic transport-plasma	9.4	1214	8	1203	7.8	1208
Manually walked-plasma	4.2	718	5.1	814	5.2	819
Manually walked-serum/whole blood	3.7	—	4.8	—	4.4	—
